# Algorithms for Screening for Active Tuberculosis among Individuals with Latent Tuberculosis Infection in a Rural Community in China

**DOI:** 10.1128/spectrum.02967-22

**Published:** 2022-11-29

**Authors:** Henan Xin, Ying Du, Xuefang Cao, Dakuan Wang, Bin Zhang, Haoran Zhang, Boxuan Feng, Yijun He, Yongpeng He, Zhusheng Quan, Zisen Liu, Jiaoxia Yan, Ling Guan, Xueling Guan, Fei Shen, Jianmin Liu, Qi Jin, Shouguo Pan, Lei Gao

**Affiliations:** a NHC Key Laboratory of Systems Biology of Pathogens, Institute of Pathogen Biology, Chinese Academy of Medical Sciences, Beijing, People’s Republic of China; b Peking Union Medical College, Beijing, People’s Republic of China; c Center for Diseases Control and Prevention of Zhongmu, Zhengzhou, People’s Republic of China; d The Sixth People’s Hospital of Zhengzhou, Zhengzhou, People’s Republic of China; Shenzhen University School of Medicine

**Keywords:** tuberculosis, latent tuberculosis infection, active case screening, algorithms, Xpert MTB/RIF assay

## Abstract

Screening for active tuberculosis (TB) among individuals with latent tuberculosis infection (LTBI) is important for the initiation and evaluation of TB preventive treatment. The performances of different tools and their combinations had rarely been studied in community-level screening among individuals with LTBI in China. This study aimed to explore appropriate algorithms for screening for active TB among individuals with LTBI in rural China. Three sputum samples were collected from each participant for smear microscopy, culture, and an Xpert MTB/RIF assay. Chest digital radiography and TB symptoms were investigated as well. The performances of different testing algorithms were compared with that of sputum culture as the gold standard. Overall, 1,564 study participants with LTBI were investigated, with a final diagnosis of 20 TB cases by sputum culture. Compared with other tests, the Xpert MTB/RIF assay detected 80.00% (95% confidence interval [CI], 58.40% to 91.93%) of culture-positive cases, with the highest sensitivity. When tests were combined using “or,” “and,” or “step” algorithms, the highest sensitivity reached 90.00% (95% CI, 69.90% to 97.21%) for the combination of the Xpert MTB/RIF assay and chest radiography, but the positive predictive value (PPV) decreased to 22.22% (95% CI, 14.54% to 32.41%). The Xpert MTB/RIF assay alone showed the best agreement with sputum culture, with a kappa value of 0.840. Pathogen molecular detection alone showed good performance compared to the other algorithms, for ruling out active TB in general LTBI, but the high cost might be a challenge for scaling it up. Identifying those with a high risk for progression to TB more precisely and establishing a cost-effective screening algorithm deserve further exploration.

**IMPORTANCE** Enhancing community-wide active case screening in target LTBI populations is important for achieving the early treatment of active TB, and ruling active TB out is a prerequisite for initiating preventive treatment. The current study evaluated the performances of multiple tests and their combinations in screening for active TB among individuals with LTBI at the community level. Compared with the classical “TB symptoms and chest radiography” algorithm, the application of Xpert MTB/RIF improved the sensitivity from 45% to 80%. When the Xpert MTB/RIF assay was combined with chest radiography, the sensitivity was further improved to 90.00%, which achieved the World Health Organization (WHO) target product profiles. However, the algorithm requires caution as the PPV decreased from 88.89% for Xpert MTB/RIF alone to 22.22% for the combination. Xpert MTB/RIF alone offered remarkable sensitivity without compromising the PPV but would have major resource implications. Thus, identifying target populations for LTBI treatment more precisely and developing cost-effective and high-throughput screening tools and algorithms deserve further efforts.

## INTRODUCTION

Although coronavirus disease 2019 (COVID-19) has overtaken tuberculosis (TB) as the deadliest communicable disease worldwide within the past year, TB remains a global public health problem, with an estimated 10 million new cases and 1.5 million deaths in 2020. The World Health Organization (WHO) developed the End TB Strategy in 2015 aiming to halt the global TB epidemic, including the goal of a 90% reduction in incidence by 2035 (1). However, in the past 2 decades, the average rate of decline in the TB incidence rate was only 1.7% globally (2). One of the challenges is that one-fourth of the world’s population are infected with Mycobacterium tuberculosis, and 5% to 10% of them might develop active TB in their lifetime (1). The implementation of preventive treatment among individuals with a high likelihood of developing active disease from latent tuberculosis infection (LTBI) has been considered one of the critical components of the End TB Strategy. In consideration of the possible risk of drug resistance, active TB cases must be ruled out before the initiation of preventive treatment to avoid the misclassification of intervention targets. In addition, strengthening screening for active TB among individuals with LTBI is also a potential tool to support the early diagnosis and early treatment of active TB (3, 4). It is well known that TB screening should consider multiple factors, including the TB epidemic in target populations, the accuracy and yield of the diagnostic methods, and the cost, availability, and feasibility of the strategy. Currently, TB symptom investigation and chest radiography are the most frequently used screening tools, as recommended by the WHO (5). However, the unsatisfactory sensitivity and specificity of TB symptoms and chest radiography need to be considered. It was estimated that 7 million people are currently living with subclinical TB without developing symptoms (6). Case detection relies on patient self-reporting of symptoms, leaving 40% of estimated TB cases undiagnosed (7). Regarding chest radiography, abnormal chest radiographic findings are not TB specific; other pulmonary diseases might present similar radiographic lesions, and as 70% of TB cases occur in rural China (8), the ability to identify TB cases by radiologists from primary-level health institutions is limited.

Considering the good performance of the Xpert MTB/RIF assay as a diagnostic tool (9–11), molecular rapid diagnostic tests were first recommended together with symptom screening and chest radiography, alone or in combination, as screening tools for the general population and high-risk groups in the most recent consolidated guidelines on tuberculosis by the WHO (12). However, the inferences need more supporting evidence, and with respect to feasibility and the resources available, the WHO suggested prioritizing TB screening using molecular rapid diagnostic tests for certain subpopulations with a higher risk of TB. In high-burden countries like China, focusing only on high-risk subgroups such as those with HIV infection or close contacts alone may not make an obvious contribution to the decline in the TB incidence in the entire community. Scaling up active case finding from high-risk subgroups to key populations in the community is one important component of the comanagement of TB and LTBI. Previously, we implemented two community-based randomized controlled trials (RCTs) aiming to explore short-course LTBI treatment regimens among individuals aged 50 to 70 years (13) and those with fibrotic chest radiography abnormalities (our unpublished data). During follow-up investigations, the Xpert MTB/RIF assay, chest radiography, smear microscopy, sputum culture, and TB symptom investigation were used simultaneously to track the incidence of active TB cases. This provided us a unique opportunity to explore whether the Xpert MTB/RIF assay could be used as an initial screening tool, and we retrospectively compared and evaluated the performances of multiple tests (alone or in combination) for TB screening among target individuals with LTBI in a rural community.

## RESULTS

### Participant enrollment and test results.

**FIG 1:**
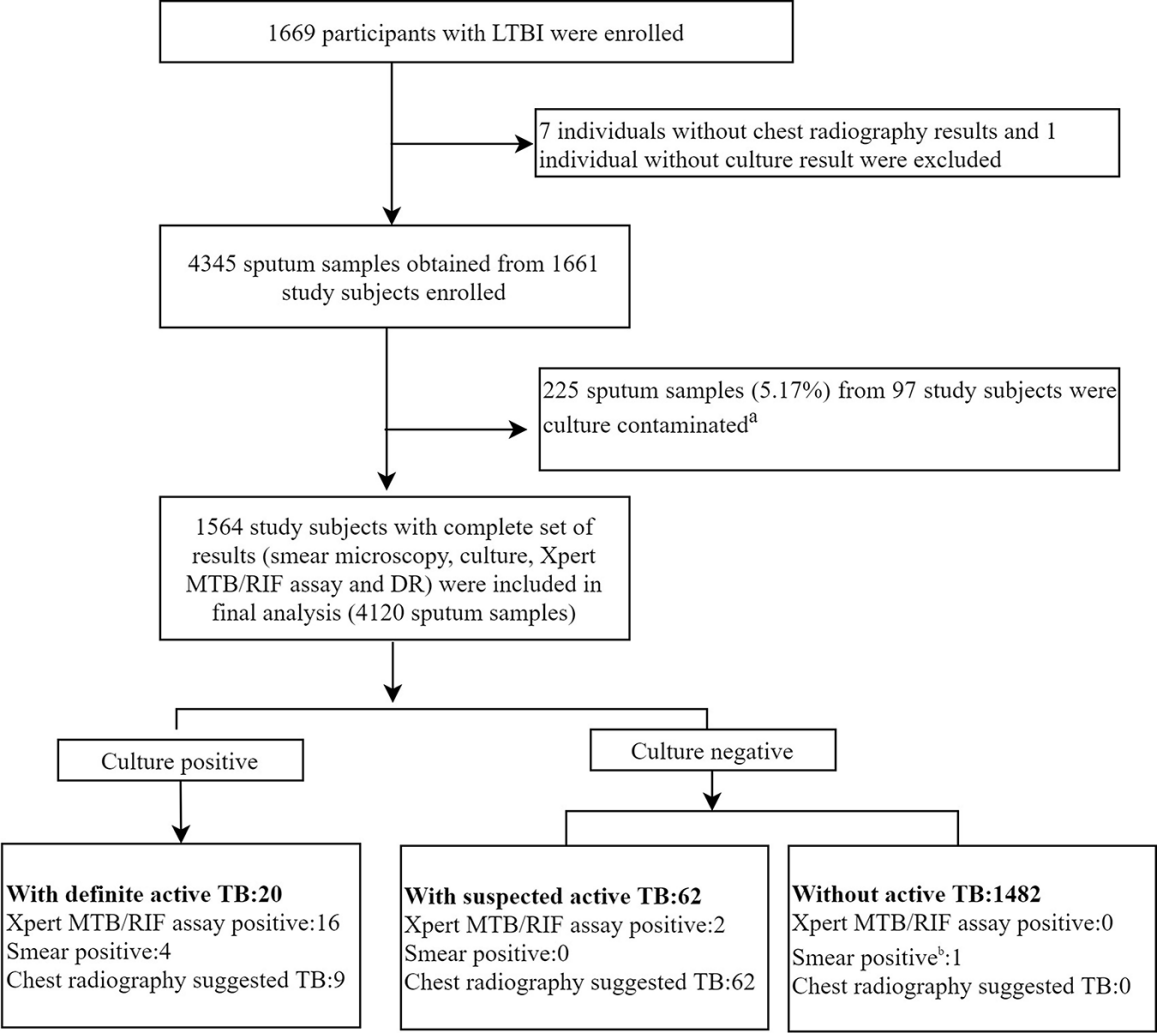
Flowchart outlining participant enrollment and outcomes stratified by diagnostic category. Definite TB is defined as individuals with culture-based evidence of MTBC. Suggested active TB is defined as individuals with a clinical-radiological picture highly suggestive of TB as determined by local senior radiographers but who did not have culture-based evidence of MTBC. Without active TB is defined as individuals with no evidence of TB based on culture and no radiological evidence to support the diagnosis of active TB. ^a^Two patients who had contaminated culture results were Xpert MTB/RIF positive. ^b^One patient was culture positive for NTM.

In total, 1,669 participants with LTBI (1,182 from trial 1 and 487 from trial 2) were investigated for active TB by sputum examination, chest radiography, and symptom investigation. After excluding 7 individuals without chest radiography results and 1 individual without culture result, 4,345 sputum samples from 1,661 participants were finally tested. The results of culture and the Xpert MTB/RIF assay for 1,661 participants are shown in Table 1. There were 20 (1.20%) and 30 (1.81%) subjects with positive results from solid medium that were identified as the M. tuberculosis complex (MTBC) and nontuberculous mycobacteria (NTM) by sequencing, respectively. Among 20 individuals (1.20%) with positive Xpert MTB/RIF results (3 at the medium level, 2 at the low level, and 15 at the very low level), 2 were culture negative and 2 had contaminated cultures. After excluding 225 sputum samples (5.17%) from 97 participants with culture contamination, 1,564 study subjects with a complete set of results were included in the final analysis (Fig. 1). Twenty subjects were reported to have definite TB, and 16 (80.00%), 4 (20.00%), and 9 (45.00%) of them were Xpert MTB/RIF positive, were smear positive, and had chest radiography abnormalities consistent with active TB, respectively. A total of 62 subjects (2 of them were Xpert MTB/RIF positive) were classified as having suspected active TB by chest radiography. The remaining 1,482 subjects were classified as being without active TB.

**TABLE 1:** Results of culture and the Xpert MTB/RIF assay for 1,661 study participants^*a*^

Xpert MTB/RIF assay result	No. of participants with culture result (%)				
	MTBC	NTM	Negative	Contaminated	Total
Medium	3	0	0	0	3 (0.18)
Low	2	0	0	0	2 (0.12)
Very low	11	0	2	2	15 (0.90)
Negative	4	30	1,512	95	1,641 (98.80)

Total	20 (1.20)	30 (1.81)	1,514 (91.15)	97 (5.84)	1,661 (100)

a MTBC, Mycobacterium tuberculosis complex; NTM, nontuberculous mycobacteria.

### Characteristics of the study participants.

The characteristics of the study participants are displayed in Table 2. For the 1,564 study participants finally included, the median age was 65 years old, and nearly 60% (911/1,564) of them were male. Approximately 30% (468/1,564) and 40% (605/1,564) had drinking and smoking habits, respectively. In addition, 9.87% (153/1,550) and 12.28% (192/1,564) of the study participants reported a history of diabetes and prior TB, respectively. When considering the subjects without active TB as controls, older individuals and those with a lower body mass index (BMI) were observed to be more likely to be diagnosed with definite TB or suspected TB.

**TABLE 2:** Characteristics of 1,564 study participants classified by diagnostic category^*e*^

Variable	Value for group				*P* value for definite active vs without active TB^*a*^	*P* value for suspected active TB vs without active TB^*b*^
	Overall	Without active TB	With definite active TB	With suspected active TB		
Total no. of participants (%)	1,564 (100)	1,482 (100)	20 (100)	62 (100)		
No. of participants of gender (%)						
Male	911 (58.25)	852 (57.49)	15 (75.00)	44 (70.97)	0.115	0.035
Female	653 (41.75)	630 (42.51)	5 (25.00)	18 (29.03)		
No. of participants of age (yrs) (%)						
≤65	847 (54.16)	821 (55.40)	5 (25.00)	21 (33.87)	0.007	0.001
>65	717 (45.84)	661 (44.60)	15 (75.00)	41 (66.13)		
Median age (yrs) (IQR)	65 (58–70)	65 (58–70)	71 (65–74)	68 (61–72)		
No. of participants with BMI (kg/m^2^) (%)						
<18.5	37 (2.37)	32 (2.16)	0 (0.00)	5 (8.06)	0.004	0.003
≥18.5–<24.0	616 (39.39)	572 (38.60)	15 (75.00)	29 (46.77)		
≥24.0	911 (58.25)	878 (59.24)	5 (25.00)	28 (45.16)		
No. of participants with alcohol use (%)						
No	1,096 (70.08)	1,042 (70.31)	12 (60.00)	42 (67.74)	0.317	0.665
Yes	468 (29.92)	540 (29.69)	8 (40.00)	20 (32.26)		
No. of participants with smoking status (%)						
Never smoked	959 (61.32)	924 (62.35)	11 (55.00)	24 (38.71)	0.500	<0.001
Ever smoked	605 (38.68)	558 (37.65)	9 (45.00)	38 (61.29)		
No. of participants with diabetes (%)^*c*^						
No	1,397 (90.13)	1,323 (90.06)	18 (90.00)	56 (91.80)	1.000	0.655
Yes	153 (9.87)	146 (9.94)	2 (10.00)	5 (8.20)		
No. of participants with prior TB history (%)^*d*^						
No	1,372 (87.72)	1,317 (88.87)	19 (95.00)	36 (58.06)	0.716	<0.001
Yes	192 (12.28)	165 (11.13)	1 (5.00)	26 (41.94)		

a *P* values by χ^2^ tests were used to compare categorical variables between individuals with definite active TB and individuals without active TB. A *P* value of <0.025 was considered statistically significant.

b *P* values by χ^2^ tests were used to compare categorical variables between individuals with suspected active TB and individuals without active TB. A *P* value of <0.025 was considered statistically significant.

c Self-reported history of diabetes or a fasting blood glucose level of >7 mmol/L.

d Self-reported history of TB who had been diagnosed before 2016.

e BMI, body mass index; IQR, interquartile range; TB, tuberculosis. The sum might not equal the total because of missing data.

Results of association analyses for TB incidence are shown in Table S1 in the supplemental material. Individuals aged >65 years old showed an increased risk of active TB compared to those who were ≤65 years old, with an adjusted odds ratio (OR) of 3.50 (95% confidence interval [CI], 1.26 to 9.72), while for individuals with a BMI of ≥24.0 kg/m^2^, their risk of acquiring active TB decreased by 76% compared to those with a BMI of <24.0 kg/m^2^.

### Performances of different testing algorithms.

The performances of different testing algorithms (smear microscopy, Xpert MTB/RIF assay, chest radiography, and TB symptoms, used alone or in combination) were compared with that of culture, as shown in Table 3. When used alone, the lowest sensitivity was observed for TB symptoms (5.00% [95% CI, 0.88% to 23.61%]), followed by smear microscopy (20.00% [95% CI, 8.07% to 41.60%]), chest radiography (45.00% [95% CI, 25.82% to 65.79%]), and Xpert MTB/RIF (80.00% [95% CI, 58.40% to 91.93%]). When different tests were combined using “or” or “and” algorithms, the best sensitivities were observed for “chest radiography positive or Xpert MTB/RIF positive” and “smear microscopy positive, chest radiography positive, or Xpert MTB/RIF positive,” with identical sensitivities (90.00% [95% CI, 69.90% to 97.21%]), while the positive predictive value (PPV) decreased to 22% for the two combinations. The sensitivity of the WHO-recommended algorithm was 45% (95% CI, 25.82% to 65.79%). The overall specificity for different diagnosis algorithms ranged from 95.92% (95% CI, 94.81% to 96.80%) to 99.94% (95% CI, 99.63% to 99.99%). When different tests were combined using “step” algorithms, the sensitivity ranged from 35% (95% CI, 18.12% to 56.17%) for “chest radiography or symptom positive followed by an Xpert MTB/RIF assay” to 80% (95% CI, 58.40% to 91.93%) for “smear microscopy negative followed by an Xpert MTB/RIF assay,” with the corresponding PPV ranging from 77.78% (95% CI, 45.26% to 93.68%) to 84.24% (95% CI, 62.44% to 94.48%). When the sensitivity, specificity, PPV, and negative predictive value (NPV) were taken into account, Xpert MTB/RIF alone showed the best performance, with a kappa value of 0.840. Sensitivity analyses were conducted after excluding those participants with prior TB history, and the results were slightly improved (Table S2).

**TABLE 3:** Diagnostic test performances of smear microscopy, the Xpert MTB/RIF assay, digital radiography, and TB symptoms^*d*^

Test or result	Performance (%) (95% CI), no. of positive participants/total no. of participants tested				Cohen’s κ
	Sensitivity	Specificity	PPV	NPV	
Smear microscopy	20.00 (8.07–41.60), 4/20	99.94 (99.63–99.99), 1,543/1,544	80.00 (37.56–96.38), 4/5	98.97 (98.34–99.37), 1,543/1,559	0.317
Xpert MTB/RIF	80.00 (58.40–91.93), 16/20	99.87 (99.53–99.96), 1,542/1,544	88.89 (67.20–96.90), 16/18	99.74 (99.34–99.90), 1,542/1,546	0.840
Chest radiography	45.00 (25.82–65.79), 9/20	95.98 (94.89–96.85), 1,482/1,544	12.68 (6.82–22.37), 9/71	99.26 (98.69–99.59), 1,482/1,493	0.182
TB symptoms	5.00 (0.88–23.61), 1/20	98.83 (98.16–99.26), 1,526/1,544	5.26 (0.93–24.64), 1/19	98.77 (98.09–99.21), 1,526/1,545	0.039
TB symptom positive or chest radiography positive^*a*^	45.00 (25.82–65.79), 9/20	95.92 (94.81–96.80), 1,481/1,544	12.50 (6.72–22.08), 9/72	99.26 (98.68–99.59), 1,481/1,492	0.179
Smear microscopy positive or Xpert MTB/RIF assay positive	80.00 (58.40–91.93), 16/20	99.81 (99.43–99.93), 1,541/1,544	84.21 (62.44–94.48), 16/19	99.74 (99.34–99.90), 1,541/1,545	0.818
Smear microscopy positive or chest radiography positive	60.00 (38.66–78.12), 12/20	95.92 (94.81–96.80), 1,481/1,544	16.00 (9.40–25.92), 12/75	99.46 (98.94–99.73), 1,481/1,489	0.237
Xpert MTB/RIF positive or chest radiography positive	90.00 (69.90–97.21), 18/20	95.98 (94.89–96.85), 1,482/1,544	22.50 (14.73–32.79), 18/80	99.87 (99.51–99.96), 1,482/1,484	0.347
Smear microscopy positive, Xpert MTB/RIF assay positive, or chest radiography positive	90.00 (69.90–97.21), 18/20	95.92 (94.81–96.80), 1,481/1,544	22.22 (14.54–32.41), 18/81	99.87 (99.51–99.96), 1,481/1,483	0.343
Smear microscopy followed by Xpert MTB/RIF^*b*^	80.00 (58.40–91.93), 16/20	99.81 (99.43–99.93), 1,541/1,544	84.24 (62.44–94.48), 16/19	99.74 (99.34–99.90), 1,541/1,545	0.818
Smear microscopy followed by chest radiography and symptoms^*b*^ followed by Xpert MTB/RIF assay^*c*^	50.00 (29.93–70.07), 10/20	99.81 (99.43–99.93), 1,541/1,544	76.92 (49.75–91.82), 10/13	99.36 (98.82–99.65), 1,541/1,551	0.602
Chest radiography and symptoms followed by Xpert MTB/RIF assay^*c*^	35.00 (18.12–56.17), 7/20	99.87 (99.53–99.96), 1,542/1,544	77.78 (45.26–93.68), 7/9	99.16 (98.57–99.51), 1,542/1,555	0.479
Chest radiography and symptoms followed by smear microscopy^*c*^ followed by Xpert MTB/RIF assay^*b*^	35.00 (18.12–56.17), 7/20	99.87 (99.53–99.96), 1,542/1,544	77.78 (45.26–93.68), 7/9	99.16 (98.57–99.51), 1,542/1,555	0.479

a WHO-recommended algorithm.

b Performed if smear negative.

c Performed if digital radiography results were compatible with active TB or TB symptoms.

d CI, confidence interval; PPV, positive predictive value; NPV, negative predictive value.

### Characteristics of study participants with discordant results for culture and the Xpert MTB/RIF assay.

As shown in Table 4, two Xpert MTB/RIF-positive subjects were culture negative. Subject 1 had been diagnosed with TB in March 2014. Both of the subjects had chest radiography results that were highly suggestive of TB, but neither of them accepted anti-TB treatment. Six months later, we retested their sputum samples by Xpert MTB/RIF: subject 1 converted to a negative result, and both of their culture results were negative. Samples from two Xpert MTB/RIF-positive subjects were culture contaminated. Subject 3 was retested 6 months later and was both Xpert MTB/RIF and culture negative. Subject 4 was diagnosed with a pulmonary tumor, and no follow-up results were available.

**TABLE 4:** Demographic information for 8 subjects with discordant results of culture and the Xpert MTB/RIF assay^*a*^

Test, result, and subject	Gender	Age (yrs)	Previous TB history	Current DR result	Grade
Xpert MTB/RIF					
Xpert MTB/RIF positive and culture negative (2 subjects)					
Subject 1	Male	71	Yes	Radiographic abnormality suggestive of active TB	Extremely low
Subject 2	Male	68	No	Radiographic abnormality suggestive of active TB	Extremely low
Xpert MTB/RIF positive and culture contaminated (2 subjects)					
Subject 3	Female	65	No	Normal	Extremely low
Subject 4	Male	66	No	Radiographic abnormality suggestive of active TB	Extremely low
Culture					
Xpert MTB/RIF negative and culture positive (4 subjects)					
Subject 5	Male	73	Yes	Radiographic abnormality suggestive of prior TB	1+
Subject 6	Male	69	No	Radiographic abnormality suggestive of active TB	1
Subject 7	Male	63	No	Radiographic abnormality suggestive of active TB	2+
Subject 8	Female	63	No	Radiographic abnormality suggestive of prior TB	4

a DR, digital radiography; TB, tuberculosis.

Four culture-positive subjects were Xpert MTB/RIF negative. One of them had been diagnosed with TB in July 2012.

## DISCUSSION

As far as we know, this is the first study from China to evaluate the performances of multiple tests for improving the TB diagnostic capacity for individuals with LTBI from a rural community. A total of 1,564 study participants were included in the study, and 20 TB cases were identified using sputum culture as the gold standard for diagnosis. Compared with the other tests, Xpert MTB/RIF detected 80.00% of culture-positive cases, with the highest sensitivity. When different tests were combined using “or,” “and,” or “step” algorithms, the classical “TB symptoms and chest radiography” algorithm has a suboptimal sensitivity of 45%. The highest sensitivity reached 90.00% for the combination of Xpert MTB/RIF and chest radiography, which reached the WHO-set minimal target of product profiles, but the PPV decreased to 22.22%. Comprehensively, Xpert MTB/RIF alone showed the best agreement with sputum culture, with a kappa value of 0.840.

In view of its ease of implementation, low cost, and rapid, simple, and high acceptability, the algorithm of “TB symptoms and chest radiographic findings” was still the dominant tool for TB screening. However, the classical algorithm has low sensitivity, which might be partly explained by the existence of subclinical TB. Thus, how to accurately rule out active TB, especially subclinical TB, before intervention is one of the concerns in LTBI management. The advent of molecular detection helps tackle this problem. In the current study, the application of Xpert MTB/RIF improved the sensitivity from 45% to 80%. When the Xpert MTB/RIF assay was combined with chest radiography, the sensitivity improved to 90.00%, which achieved the WHO target product profiles, indicating that chest radiography can make up for the drawbacks of poor sputum quality using Xpert MTB/RIF and might be a useful tool for community screening (14, 15). However, the algorithm requires caution as the PPV decreased from 88.89% for Xpert MTB/RIF alone to 22.22% for the combination. Chest radiography might overestimate the number of bacterium-negative TB cases (16). The low prevalence of TB in study participants and the suboptimal specificity of chest radiography might account for the decreased PPV. Xpert MTB/RIF alone for all samples offered remarkable sensitivity gains without compromising the PPV but would have major resource implications and the need for additional equipment since the minimum purchase price of the Xpert MTB/RIF assay is about $40 per person. The classical “initial TB symptoms or chest radiography with a subsequent microbiological test among those who are positive” algorithm might be preferred for TB screening in a general population with a low prevalence of the disease considering cost-effectiveness. But with respect to individuals with LTBI, who contribute significantly to the pool of active TB cases in countries with a high burden of TB (17), TB screening would not only contribute to early case finding but also help to avoid misclassifying current cases as targets for preventive treatment. Thus, considering the target population for screening and the comanagement of LTBI and TB, balancing the performance of the tests with the availability, feasibility, and costs of the tests, including LTBI testing, is a prerequisite for achieving precise intervention in countries with a high burden of TB, like China. Identifying target populations with a high risk of developing TB from LTBI more precisely is one solution. Blood-based, host-derived immune response biomarkers that provide a broad view of the host response to TB have shown considerable promise and have been explored to guide preventive treatment (18, 19). Furthermore, accelerating the development and application of other cutting-edge diagnostic technologies with high throughput and low costs, such as nucleic acid mass spectrometry or artificial intelligence, is another solution. It was previously reported that computer-aided detection software could be highly accurate and useful triage tools for TB detection in high-burden regions and outperformed human readers (20), and it was first recommended in the most recent guidelines (12); whether it could play an important role in future TB screening needs further evidence.

Although culture is the gold standard for the diagnosis of TB, its long turnaround time renders it useless for rapid diagnosis, while as an automated, rapid molecular diagnostic method, the Xpert MTB/RIF assay can generate results within 2 h. Due to its high sensitivity and short turnaround time, Xpert MTB/RIF can play a crucial role in improving TB case detection. A meta-analysis including 9,557 participants from 27 studies summarized the sensitivity of Xpert MTB/RIF as 89% (95% CI, 85% to 92%), which was higher than our estimate (80% [95% CI, 58% to 92%]) (21). However, their study participants were presumed to have pulmonary TB, drug-resistant TB (DR-TB), or HIV infection, which might explain the difference. Previous studies using Xpert MTB/RIF as an initial tool focused mainly on HIV infections (9, 10). There have been few studies that have evaluated the performance of the “Xpert MTB/RIF for all” algorithm in community-wide active case finding. Although 43,435 adults consented to screening with Xpert MTB/RIF in a previous study by J. Ho et al. (22), those researchers were unable to estimate test sensitivity as culture was not done with samples from all screened participants. Our study provides preliminary data on the performance of Xpert MTB/RIF among general at-risk target populations with LTBI.

For the four subjects with Xpert MTB/RIF-positive but culture-negative results, two of them reverted to Xpert MTB/RIF negative after 6 months, and one of them was a previously treated TB patient. Our results are in line with those of a previous study by G. Theron et al., who retested patients with previous TB using Xpert MTB/RIF. In this study, 9 of 15 Xpert MTB/RIF-positive, culture-negative patients (“Xpert MTB/RIF false positive”) reverted to Xpert MTB/RIF negative after 2 to 3 months (23). All of them were clinically well, without treatment after follow-up. The results from a prospective follow-up indicated that some of the Xpert MTB/RIF false positives might be true Xpert MTB/RIF false positives that were confounded by residual mycobacterial DNA from dead bacilli. The slightly improved performance of our results after excluding subjects who had a history of prior TB further verified the above-mentioned hypothesis. Despite this, the existence of subclinical TB cannot be fully excluded as most subjects were asymptomatic. The Xpert MTB/RIF retesting result for subject 2 was still positive after 6 months. Subject 2 reported a history of close contact with active TB patients, but he had never been diagnosed with active TB. Unfortunately, as subject 2 refused anti-TB treatment, we cannot verify our hypothesis through his responses to treatment.

There were several limitations of this study. First, as the study participants were target populations from two RCTs, the generalizability of our findings is limited. Second, considering that the majority of participants lacked TB symptoms, two slopes were cultured for each sputum sample (six slopes for each person) to increase the detection rate. In addition, induced spot sputum was collected as it was previously reported that it could increase TB case detection for individuals who were unable to expectorate spontaneously (24). Although multiple methods were used to increase the sensitivity of testing, we cannot completely exclude that several TB cases, especially subclinical TB cases, might be underdiagnosed because of sputum-scarce samples. Third, although the Xpert MTB/RIF Ultra assay had a huge improvement in sensitivity and is currently widely used abroad (25), it was not yet available in China when the trials were done. In addition, the Xpert MTB/RIF assay might be more suitable considering the compromised specificity of the Xpert MTB/RIF Ultra assay for individuals with previous TB (26). Finally, we did not evaluate the performance of the Xpert MTB/RIF assay in defining DR-TB as analyses of the results between phenotypic drug susceptibility testing and genotypic Xpert MTB/RIF were unavailable.

### Conclusions.

Enhancing community-wide active case screening in target LTBI populations is important for achieving the early treatment of active TB, ruling out active disease before LTBI treatment, and, finally, achieving comprehensive management of TB and LTBI. Our results indicated that the Xpert MTB/RIF assay alone showed better agreement with sputum culture than the other studied tools alone or in combination. This consistently suggested that pathogen molecular testing could be a more precise and faster tool for screening for active TB. However, the cost might be a challenge for promoting its application in regions with limited resources. Therefore, identifying target populations for LTBI treatment more precisely and developing cost-effective and high-throughput screening tools and algorithms deserve further effort, which is essential for promoting LTBI management as well as TB control worldwide.

## MATERIALS AND METHODS

### Study design and population.

In 2015 and 2018, we implemented two community-based, RCTs aiming to screen individuals with LTBI and explore short-course LTBI treatment regimens. The current study participants came from the untreated control groups of the two trials (the two RCTs were registered at the Chinese Clinical Trial Registry with the identifiers ChiCTR-IOR-15007202 and ChiCTR-1800018224). Trial 1 focused on general elderly Chinese individuals (50 to 70 years old) in rural communities, and trial 2 targeted individuals with fibrotic radiological lesions aged 18 to 70 years from rural communities. LTBI was diagnosed with the QuantiFERON-TB gold in-tube (QFT-GIT) assay according to the manufacturer’s instructions, with a cutoff value of ≥0.35 IU/mL. In August 2020, microbiological assays (sputum culture, smear microscopy, and Xpert MTB/RIF), chest radiography, and TB symptom investigations were conducted among the study participants during a follow-up investigation in order to improve the TB diagnosis and find more TB cases.

### Procedures.

**(i) Sputum sample collection.** Three sputum samples (one night sputum, one morning sputum, and one spot sputum, with at least 2 mL for each sputum sample) were collected from each participant involved in the follow-up survey. Participants were guided on how to expectorate a deep sputum specimen and were provided two sputum boxes for night sputum and morning sputum sample collection the day before the examination. On the day of the examination, participants were required to expectorate one spot specimen by inhaling 5% sodium chloride for 10 min. For those without night sputum or morning sputum samples, the induced spot sputum sample must have been available.

**(ii) Laboratory tests.** Each of the three sputum samples was processed for sputum microscopy after Ziehl-Neelsen staining. For sputum culture, a 1-mL sample was processed with 2 to 3 mL 4% sodium hydroxide for 15 min, followed by centrifugation. Next, two slopes were cultured for each of the three sputum samples on solid Lowenstein-Jensen medium. The identification of the MTBC was confirmed by sequencing using a 16S rRNA gene sequencing system. The Xpert MTB/RIF assay (Cepheid, Sunnyvale, CA, USA) was conducted and interpreted according to the manufacturer’s recommendations. One-milliliter sputum samples were drawn from each of the three sputum samples and fully mixed. Next, 1-mL sputum samples were extracted from the 3-mL mixture, added to the Xpert MTB/RIF kit sample reagent in a 1:2 (vol/vol) ratio, and incubated at room temperature for 15 min, and 2 mL of this mixture was added to an Xpert MTB/RIF cartridge. If there were fewer than 3 samples, 1 or 2 specimens were used or mixed for Xpert MTB/RIF. A 16-module GeneXpert instrument with an automated readout was used. Repeat Xpert MTB/RIF assays using the same sample were conducted if invalid results occurred. A smear-positive TB case was defined as having at least 1 acid-fast bacillus in at least 1 sputum sample. Culture positivity was defined as a positive result from solid medium identified as MTBC by sequencing. NTM were defined as a positive result from solid medium culture identified as NTM by sequencing.

**(iii) Chest radiographs.** All participants were investigated by a questionnaire for TB symptoms (cough for longer than 2 weeks with sputum production, which may have blood at times) and examined by chest radiography. Radiographic abnormalities consistent with active TB were diagnosed by a team of three radiologists.

### TB case definitions.

Each participant was allocated to one of three diagnostic categories: (i) definite TB, with at least one slope culture positive for MTBC; (ii) suspected active TB, with a clinical-radiological picture highly suggestive of TB as determined by radiographers regardless of TB symptoms but without culture-based evidence of MTBC; and (iii) without active TB, with no evidence of TB based on culture and no radiological evidence to support the diagnosis of active TB.

### Statistical analyses.

All analyses were done using SAS 9.4 (SAS Institute Inc., NC, USA). Pearson’s χ^2^ tests were used to compare the categorical variables. In the multivariable logistic regression analyses, variables with *P* values of less than 0.05 in the univariate models were included in the model with age and sex fixed in the models as well. The associations were estimated by the OR and 95% CI. A two-tailed test statistic was considered significant at a *P* value of ≤0.05. If multiple-comparison testing was needed, the level of significance was adjusted using the Bonferroni method by dividing the significance level of 0.05 by the number of simultaneous tests. Different tests were used alone or combined using “or,” “and,” or “step” to generate different algorithms. The sensitivity, specificity, PPV, and NPV with 95% CI were calculated using sputum culture as the gold standard to evaluate the performances of different algorithms. Individuals who were culture negative for all samples (i.e., those with suspected active TB and those without active TB) were used for specificity calculations. Kappa values were used to evaluate the agreement between different screening methods and sputum culture.

### Ethics approval.

Written informed consent was obtained from each patient before participation, and the study was conducted in accordance with the Declaration of Helsinki. The ethics committees of the Institute of Pathogen Biology and the Chinese Academy of Medical Sciences approved the study protocol (IPB-2020-05).

### Data availability.

We confirm that the data supporting the findings of this study are available within the article and its supplemental materials.
