# Supplementary material for: Algorithms for Screening for Active Tuberculosis among Individuals with Latent Tuberculosis Infection in a Rural Community in China
Source: Microbiol Spectr. 2022 Nov 29;10(6):e02967-22. doi: 10.1128/spectrum.02967-22 (PMC9769587; doi:10.1128/spectrum.02967-22)
Supplement: Supplemental file 1 — Supplemental material. Download spectrum.02967-22-s0001.pdf, PDF file, 0.2 MB [file spectrum.02967-22-s0001.pdf]

Supporting Information

Algorithms for screening active tuberculosis among individuals with latent tuberculosis infection in rural community, China

Henan Xin1*, Ying Du1*, Xuefang Cao1*, Dakuan Wang2*, Bin Zhang2*, Haoran Zhang1, Boxuan Feng1, Yijun He1, Yongpeng He1, Zhusheng Quan1, Zisen Liu2, Jiaoxia Yan2, Ling Guan3, Xueling Guan3, Fei Shen3, Jianmin Liu3, Qi Jin1, Shouguo Pan2✉, Lei Gao1✉

1. NHC Key Laboratory of Systems Biology of Pathogens, Institute of Pathogen Biology, Chinese Academy of Medical Sciences and Peking Union Medical College, Beijing 100730, P.R. China
2. Center for Diseases Control and Prevention of Zhongmu, Zhengzhou 451450, P.R. China.
3. The Sixth People's Hospital of Zhengzhou, Zhengzhou 400060, P.R. China.

* Contributed equally

Correspondence: Prof. Lei Gao, NHC Key Laboratory of Systems Biology of Pathogens, Institute of Pathogen Biology, and Center for Tuberculosis Research, Chinese Academy of Medical Sciences and Peking Union Medical College, No 9 Dong Dan San Tiao, Beijing 100730, China. Email: gaolei@ipbcams.ac.cn. Prof. Shouguo Pan, Center for Diseases Control and Prevention of Zhongmu, No. 1106, Youth West Road, Zhongmou County, Zhengzhou 451450, China., Email: psgzmcdc@163.com.

Table S1. Association analysis for the incidence of TB disease

Variables	TB disease n/N (%)	p for $\chi2$ test	Adjusted OR (95% CI)
**Total**			
**Gender**			
Male	15/911 (1.65)	0.126	Reference
Female	5/653 (0.77)		0.49 (0.18-1.37)
**Age (years)**			
≤65	5/847 (0.59)	**0.008**	Reference
>65	15/717 (2.09)		**3.50 (1.26-9.72)**
**BMI (kg/m^2)**			
-<24.0	15/653 (2.30)	**0.002**	Reference
≥24.0	5/911 (0.55)		**0.24 (0.09-0.67)**
**Alcohol drinking**			
No	12/1096 (1.09)	0.322	
Yes	8/468 (1.71)		
**Smoke status**			
Never smoked	11/959 (1.15)	0.559	
Ever smoked	9/ 605 (1.49)		
**With diabetes** [a]			
No	18/1397 (1.29)	0.985	
Yes	2/153 (1.31)		
**With prior TB history** [b]			
No	19/1372 (1.38)	0.499	
Yes	1/192 (0.52)		

Abbreviation: BMI, body mass index; CI, confidence interval; OR, odds ratio; TB, tuberculosis.

a self-reported history of diabetes or fasting blood glucose >7mmol/L

b self-reported history of TB.

Table S2 Diagnostic test performance of smear microscopy, Xpert MTB/RIF assay, DR, and TB symptoms after excluding those with prior TB history

Various tests	Performance				
	**Sensitivity (95%CI)**	**Specificity(95%CI)**	**PPV (95%CI)**	**NPV (95%CI)**	**Cohen's κ**
Smear microscopy	21.05 (8.51-43.33)<br>4/19	99.93 (99.58-99.99)<br>1352/1353	80.00(37.56-96.38)<br>4/5	98.90 (98.20-99.33)<br>1352/1367	0.330
Xpert MTB/RIF assay	84.21 (62.44-94.48)<br>16/19	99.94 (99.58-99.99)<br>1352/1353	94.12 (73.02-98.95)<br>16/17	99.69 (99.35-99.92)<br>1352/1355	**0.887**
Chest radiography	47.37 (27.33-68.29)<br>9/19	97.33 (96.34-98.07)<br>1317/1353	20.00 (10.09-33.82)<br>9/45	99.25 (98.62-99.59)<br>1317/1327	0.267
TB symptoms	5.26 (0.95-24.64)<br>1/19	99.03 (98.36-99.44)<br>1340/1353	7.14 (1.27-31.47)<br>1/14	98.67 (97.91-99.16)<br>1340/1358	0.049
TB symptoms positive or chest radiography positive [a]	47.37 (27.33-68.29)<br>9/19	97.27 (96.25-98.01)<br>1316/1353	19.57 (10.65-33.17)<br>9/46	99.25 (98.62-99.59)<br>1316/1326	0.263
Smear microscopy positive or Xpert MTB/RIF assay positive	84.21 (62.44-94.48)<br>16/19	99.85 (99.46-99.96)<br>1351/1353	88.89 (67.20-96.90)<br>16/18	99.78 (99.35-99.92)<br>1351/1354	0.863
Smear microscopy positive or chest radiography positive	63.16 (41.04-80.85)<br>12/19	97.27 (96.03-97.71)<br>1316/1353	24.49 (14.60-38.09)<br>12/49	99.47 (98.91-99.74)<br>1316/1323	0.340
Xpert MTB/RIF assay positive or chest radiography positive	**94.74 (75.36-99.06)**<br>**18/19**	97.34 (96.10-97.76)<br>1317/1353	33.33 (22.24-46.64)<br>18/54	99.92 (99.57-99.99)<br>1317/1318	0.483
Smear microscopy positive or Xpert MTB/RIF assay positive or chest radiography positive	**94.74 (75.36-99.06)**<br>**18/19**	97.27 (96.25-98.01)<br>1316/1353	32.73 (21.81-45.90)<br>18/55	99.92 (99.57-99.99)<br>1316/1317	0.476
Smear microscopy followed by Xpert MTB/RIF assay [b]	84.21 (62.44-94.48)<br>16/19	99.85 (99.46-99.96)<br>1351/1353	88.89 (67.20-96.90)<br>16/18	99.78 (99.35-99.92)<br>1351/1354	0.863

Smear microscopy followed by chest radiography and symptoms [b] followed by Xpert MTB/RIF assay [c]	52.63 (31.71-72.67)<br>10/19	99.85 (99.46-99.96)<br>1351/1353	83.33 (55.20-95.30)<br>10/12	99.13 (98.75-99.65)<br>1351/1360	0.641
Chest radiography and symptoms followed by Xpert MTB/RIF assay [c]	36.84 (19.15-58.96)<br>7/19	99.93 (99.58-99.99)<br>1352/1353	87.50 (52.91-97.76)<br>7/8	99.12 (98.47-99.50)<br>1352/1364	0.515
Chest radiography and symptoms followed by smear microscopy [c] followed by Xpert MTB/RIF assay [b]	36.84 (19.15-58.96)<br>7/19	99.93 (99.58-99.99)<br>1352/1353	87.50 (52.91-97.76)<br>7/8	99.12 (98.47-99.50)<br>1352/1364	0.515

Abbreviation: CI, confidence interval; DR, digital radiography; PPV, positive predictive value; NPV, negative predictive value.

[a] WHO recommended algorithm

[b] performed if smear negative.

[c] performed if DR compatible with active TB or with TB symptoms.
